# Specific and non-specific effects of *Mycobacterium bovis* BCG vaccination in dairy calves

**DOI:** 10.3389/fvets.2023.1278329

**Published:** 2023-10-06

**Authors:** Catalina Contreras, Raúl Alegría-Moran, Mario Duchens, Pedro Ábalos, Renata López, Patricio Retamal

**Affiliations:** ^1^Facultad de Ciencias Veterinarias y Pecuarias, Universidad de Chile, Santiago, Chile; ^2^Escuela de Medicina Veterinaria, Sede Santiago, Facultad de Recursos Naturales y Medicina Veterinaria, Universidad Santo Tomás, Santiago, Chile; ^3^Magister en Ciencias Animales y Veterinarias, Universidad de Chile, Santiago, Chile

**Keywords:** BCG, cattle, non-specific, protection, bovine tuberculosis

## Abstract

Bovine tuberculosis (bTB) is a chronic disease mainly caused by *Mycobacterium bovis*, a zoonotic pathogen with economic significance as it leads to reduced milk and meat production, and high costs for control measures. The Bacillus Calmette-Guérin (BCG) vaccine, primarily used to prevent tuberculosis in humans, has also been studied for controlling bTB. While showing effectiveness in preventing *M. bovis* infection and disease in cattle, the BCG vaccine can induce non-specific effects on the immune system, enhancing responses to infections caused by unrelated pathogens, and also having non-specific effects on lactation. The aim of this study is to describe both the specific and non-specific effects of BCG vaccination in calves from a commercial dairy herd in central Chile. Diagnosis of *M. bovis* infection was performed through the IFNγ release assay (IGRA) using ESAT6/CFP-10 and Rv3615c antigens. The records of milk production, somatic cell count (SCC), clinical mastitis (CM) and retained placenta (RP) during the first lactation were compared between vaccinated and non-vaccinated animals. The breed (Holstein Friesian [HF] v/s HF × Swedish Red crossbred [HFSR]) and the season (warm v/s cold) were also analyzed as categorical explanatory variables. Results of IGRA showed significant differences between vaccinated and control groups, indicating a vaccine efficacy of 58.5% at 18 months post vaccination in HFSR crossbred animals. Although milk production did not vary, SCC and CM showed differences between groups, associated to the breed and the season, respectively. When analyzing CM and RP as a whole entity of disease, BCG showed protection in all but the cold season variables. Overall, the BCG vaccine induced protective specific and non-specific effects on health parameters, which may be influenced by the breed of animals and the season. These results provide new features of BCG protection, supporting initiatives for its implementation as a complementary tool in bTB control.

## Introduction

Bovine tuberculosis (bTB) is a chronic bacterial disease mainly caused by *Mycobacterium bovis* that affects a range of domestic and wild animals, including cattle, badgers, deer, and possums. Furthermore, bTB pose a public health risk, as it can be transmitted to humans through consumption of unpasteurized dairy products or inhalation of infectious aerosols (1). The disease represents a significant economic concern in many countries, as it can lead to decreased milk and meat production, as well as the loss of infected animals and the costs associated to control measures (2). Control and eradication of bTB often require a combination of strategies, including testing, culling of infected animals and movement restrictions.

The Bacillus Calmette-Guérin (BCG) is a live attenuated vaccine that was developed from an attenuated strain of *M. bovis*, existing several licensed formulations primarily used to prevent tuberculosis (TB) in humans, particularly in high-risk populations (3). The BCG vaccine has also been studied as a tool for controlling bovine tuberculosis (bTB) (4). While the vaccine is not highly effective in preventing *M. bovis* infection in cattle, it can provide protection against severe forms of the disease, reducing visible lesions, histopathological changes and the spread of infection (5, 6).

The BCG vaccine has been shown to induce non-specific effects on the immune system beyond its primary aim of protecting against tuberculosis, allowing the decrease in mortality by unrelated infectious agents (7, 8). The mechanism underlying these non-specific effects is not yet fully understood but may involve modulation of the immune system by BCG through a process known as trained immunity (9). Trained immunity is a long-lasting reprogramming of the innate immune system that enhances the response to subsequent infections, even those caused by unrelated pathogens, through changes at epigenetic and gene expression levels (10). In cattle, this phenomenon has been described at cellular level, in which BCG-stimulated monocytes increase the transcription of proinflammatory cytokines in response to *ex vivo* stimulation with *E. coli* LPS (11).

More recently, our group reported non-specific effects on lactation of animals that were vaccinated at 12 months, suggesting that BCG-induced metabolic changes can also increase milk production after first calving, under a natural transmission setting.

The aim of this work is to describe specific and non-specific effects of BCG vaccination in dairy calves, which records after first lactation were analyzed and compared between vaccinated and control groups, in a commercial dairy herd from central Chile.

## Materials and methods

### Herd

This work was carried out in a dairy farm in a rural area of the Metropolitan Region, Chile, enrolled for the study due to its high bTB prevalence (72%), as recorded within the sanitary web system of Agricultural and Livestock Service of Chile (SAG). Such prevalence was determined through the caudal fold testing with the bovine Purified Protein Derivatives (PPDB) antigen (Pronabive^®^, México City, México).

Previous to this study in the farm, the BCG vaccine had not been applied, and during the vaccination period, the herd contained Holstein Friesian (HF) and the F1 generation of HF × Swedish Red crossbred (HFSR) calves.

### Animals

Female calves up to 40 days-old were recruited in two groups, including those vaccinated subcutaneously with BCG Russia substrain (2–8 × 10^5^ colony forming units, 0,1 mL) (*n* = 145), and a control non-vaccinated group (*n* = 135), in which animals received 0,1 mL of vaccine diluent (NaCl 0,9%). The distribution in each group was randomized in every fieldwork.

From birth and throughout their whole productive cycle, recruited animals from both groups were managed similarly under routine conditions of the farm, aimed to optimize milk production.

### IFNγ release assay

Animals of the study were sampled at 18 months post-inoculation. For this, 5 mL of peripheral blood was collected in heparinized tubes (BD Vacutainer^®^, Franklin Lakes, NJ, United States), and processed during the same day after arrival at the laboratory. The blood was stimulated with ESAT-6/CFP-10 and Rv3615c peptide cocktails (5 μg/each peptide/mL), Pokeweed mitogen (6 μg/mL) (Applied Biosystems^®^Bovigam^®^) and PBS as a control solution (12).

After an incubation period at 37°C for 18 h, plasma supernatants were harvested and IFN-γ was detected using the Bovigam 2G^®^ Test Kit (Prionics AG, Tullamarine, Australia), according to manufacturer recommendations. The cut-off value for ESAT-6/CFP-10 and Rv3615c peptide cocktails (DIVA antigens) was an optical density at 450 nm difference ≥ 0.1 after subtraction of the PBS control values from either cocktail (ΔOD450).

### Data collection

Several visits were made to the farm for accessing productive and sanitary records, until 180 days after the first calving of animals, which occurred in the range of 24–30 months of age. The data was collected individually from each cow, including the identification numbers of animals (Official Individual Identification Device [DIIO] and the local number), date of birth, date of death, date of first delivery, breed, accumulated milk yields (L), monthly somatic cell counts (SCC), episodes of mastitis and other reproductive illnesses.

The raw milk of cows was collected twice daily with a milking machine (GEA Dematron 60, Germany) and a bulk tank milk (DXCE 7500, DeLaval, The Netherlands), and productive records were obtained with the CLI-Win^®^ software (Cooprinsem). Somatic cell count analyses were performed with a Fossomatic 90 (Foss Electric) according to ISO 13366-2/IDF148-2:2006, once or twice a month.

### Analysis of results

The efficacy of the vaccine (EV) was calculated using the formula EV(%) = ([Rn−Rv]/Rn) × 100 where Rn and Rv are the rates of the IGRA positives in the unvaccinated and vaccinated groups, respectively (13). This, assuming that positives were probably infected and negatives were probably non-infected individuals, since other diagnostic procedures were not developed. The proportions of IGRA positives between these groups were compared using the Fisher’s exact test.

The accumulated lactation yields at 180 days were calculated through the following formulae (14).


$$\begin{array}{l} MY=I_{0}M_{1}+I_{1}*\frac{\left(M_{1}+M_{2}\right)}{2}+I_{2}*\frac{\left(M_{2}+M_{3}\right)}{2}\\ +I_{n-1}*\frac{\left(M_{n-1}+M_{n}\right)}{2}+I_{n}M_{n} \end{array}$$

Where:

*MY*: accumulated milk production.

$M_{1}M_{2}M_{n}:$ liters of milk per day during the check day.

$I_{1}I_{2}I_{n-1}:$ days between checks.

*I*_0:_ days between the start date of the lactation period and the date of the first record.

*I*_n:_ days between the last record date and the end of the lactation period.

Differences between groups were analyzed with the ANOVA and *post hoc* Tukey’s test.

The SCC data during first 180 lactation days was transformed to a linear score using the following formula (15).


$$SCC=\log_{2}\left(\frac{SCC}{100000}\right)+3$$

The scores were analyzed for normality with Shapiro-Wilks test, and differences between groups were determined with the Kruskall-Wallis test. In this analysis, SCC counts of animals with a clinical mastitis episode were not included.

Pathologies recorded until the 180-lactation day were analyzed, which included clinical mastitis (CM) and retained placenta (RP). Diagnosis of CM was made through a daily macroscopic milk analysis, and RP was determined by a physical examination 24 h after parturition. Events of disease, but not their duration, were recorded for analysis. If a cow had two or more events of mastitis, it was considered only once. Two multivariable logistic regression model were constructed (one for CM and other for RP) to evaluate the association between registered variables with the outcomes (16). In these models, the response variable (*Y*) is dichotomous, because it can only take two values, where *Y* = 0 and *Y* = 1 represent the absence and presence of one or both pathogens in each BPS, respectively. Statistical significance was established at *p* < 0.05.

All the statistical analysis were performed with InfoStat software^®^ (version 2020).

Because both milk production and the health of the udder may show seasonality (17), the analysis of the response variables included the grouping of the data into warm (October to March) and cold months (April to September), based on the calving season (milk yields and post-partum pathologies) or the month of data collection (SCC). Additionally, two breed categories were compared: HF (*n* = 110, 57 BCG and 53 control) and HFSR (*n* = 148, 77 BCG and 71 control) (Supplementary Table S1).

## Results

Due to calf mortality, the IGRA was finally performed in 134 BCG vaccinated and 124 control animals (Table 1). In this assay, positive results were significantly different (*p* = 0.013) between vaccinated and control groups in HFSR crossbred animals only, showing a high EV% at 18 months post vaccination (Table 1). In contrast, HF animals did not show differences between groups, without evidence of protection conferred by BCG at this period. The overall calculated vaccine efficacy was 37.2% (95% CI 15.4 to 60.6) (Table 1).

**Table 1:** Interferon γ release assay (IGRA) positive animals and efficacy of the vaccine (EV%).

Breed	IGRA positive (%)			EV%
	BCG	Control	*p* value	
HF	10/57 (17.5)	8/53 (15.1)	0.73	0
HFSR	9/77 (11.7)	20/71 (28.2)	0.01	58.5
Total	19/134 (14.2)	28/124 (22.6)	0.08	37.2

HF, Holstein Friesian; HFSR, HF × Swedish Red crossbred.

Some animals were excluded from the analysis of post-partum variables, because they did not give birth (*n* = 20) or there were significant inconsistencies in their records (*n* = 2). These analyses finally included 123 BCG vaccinated and 113 control animals.

Cumulative milk production at 180 days did not show significant differences (*p* > 0.05) between BCG vaccinated and control individuals (Supplementary Figure S1). In contrast, the HFSR crossbred showed significant differences in SCC, in which vaccinated had lower counts than non-vaccinated animals (*p* = 0.04). When the season and overall data were analyzed, no differences were determined (Table 2).

**Table 2:** Somatic cell count (SCC) scores of BCG vaccinated and control animals during the first 180 lactation days.

	BCG			Control			*p* value
	*N*	Mean	SD	*N*	Mean	SD	
Season							
Warm	335	3.84	2.09	313	3.95	2.23	0.51
Cold	308	4.12	2.22	276	4.36	2.3	0.18
Breed							
HF	268	4.01	2.19	234	3.88	2.24	0.52
HFSR	375	3.97	2.04	355	4.34	2.26	0.02
Overall	643	3.97	2.10	589	4.14	2.27	0.17

N, number of records; SD, standard deviations; HF, Holstein Friesian; HFSR, HF × Swedish Red crossbred.

**Figure 1:**
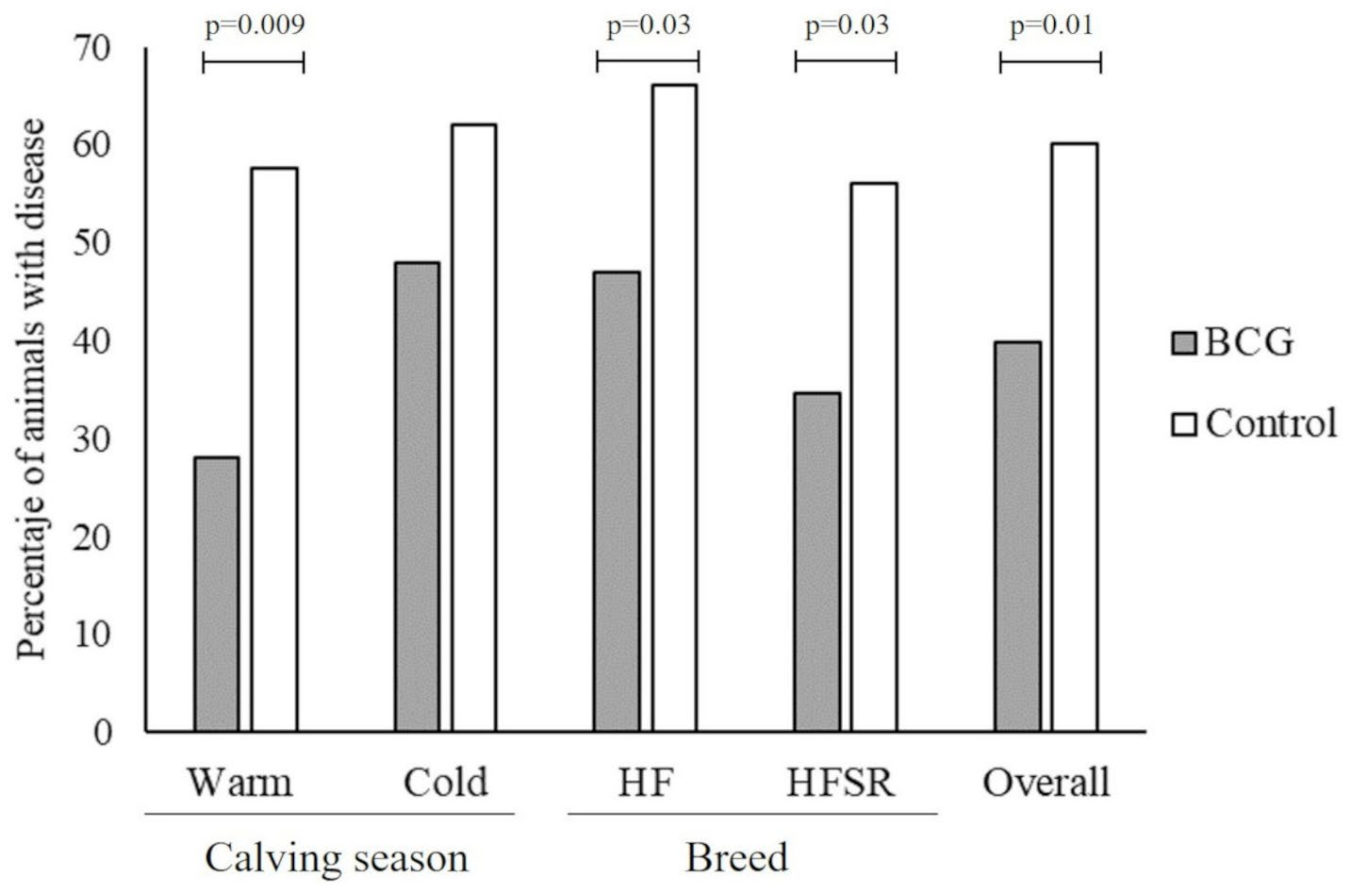
Percentage (%) of diseased animals (with clinical mastitis and/or retained placenta) within BCG vaccinated and control groups during the first 180 lactation days. The records were analyzed according to the calving season [warm (from october to march) and cold (from april to september)], the breed (HF, Holstein Friesian, and HFSR, HF × Swedish Red crossbred F1 generation), and the overall dataset.

In the analysis of retained placenta, no significant differences were found in any level of comparison (Table 3). However, in the case of clinical mastitis a distinction was observed at both the warm season and the overall level of comparison between groups (*p* = 0.04) (Table 3). When the analysis included both diseases together, all but the cold season variables showed significant differences between the vaccinated and control groups (Figure 1).

**Table 3:** Clinical mastitis and retained placenta frequencies of BCG vaccinated and control animals during the first 180 lactation days.

	BCG (%)	Control (%)	*p* value	OR (CI)
**Clinical mastitis**				
Calving season				
Warm	9/50 (18)	17/47 (36.2)	0.04	0.39 (0.15–0.98)
Cold	24/73 (32.9)	27/66 (40.9)	0.34	0.71 (0.35–1.42)
Breed				
HF	17/51 (33.3)	20/47 (42.6)	0.34	0.67 (0.29–1.53)
HFSR	16/72 (22.2)	24/66 (36.4)	0.06	0.49 (0.23–1.05)
Overall	33/123 (26.8)	44/113 (38.9)	0.04	0.57 (0.33–0.99)
**Retained placenta**				
Calving season				
Warm	5/50 (10.0)	10/47 (21.3)	0.13	0.41 (0.13–1.31)
Cold	11/73 (15.1)	14/66 (21.2)	0.35	0.66 (0.27–1.58)
Breed				
HF	7/51 (13.7)	11/47 (23.4)	0.22	0.51 (0.18–1.47)
HFSR	9/72 (12.5)	13/66 (19.7)	0.25	0.58 (0.23–1.47)
Overall	16/123 (13)	24/113 (21.2)	0.09	0.55 (0.28–1.11)

OR, odds ratio; CI, confidence interval; HF, Holstein Friesian; HFSR, HF × Swedish Red crossbred.

## Discussion

The BCG vaccine, originally developed for human use against tuberculosis, has also found application in the field of veterinary medicine, mainly for the disease prevention in both domestic and wildlife reservoirs of *Mycobacterium bovis* (18). In this context, the BCG vaccination of cattle, under diverse conditions and transmission scenarios, has shown to be a potential valuable strategy in managing and preventing bovine tuberculosis (4), but also offering non-specific improvements in animal health and productivity (12, 19).

For preventing bovine tuberculosis, BCG confers a variable protection, with an average 25% of efficacy, during a period of 12 to 24 months (4). This achievement seems to be influenced by several factors, such as vaccine strain, dose, age, health status, breed and transmission setting (18). In this study, DIVA IGRA positivity at 18 months post-vaccination was quantified as a proxy of protection, resulting the crossbred HFSR with a significant lower positivity than HF (Table 1). Although no other evidence of protection was analyzed, the IFNγ response to mycobacterial antigens ESAT-6, CFP-10 and Rv3615c has been recognized specific and sensitive for detection of *M. bovis* infection in BCG vaccinated animals (20, 21). In the study, the IFNγ response suggests 58.5% vaccine efficacy in HFSR, a result that highlights the significance of the host genetic background, in this case the breed, as a crucial factor that influences the BCG-induced immunity (22), which should be analyzed when evaluating vaccines for tuberculosis in cattle.

The goal of breeding crossbred animals is to enhance fertility and optimize health-related indicators, so that the economic decrease associated with lower milk production is accompanied by improvements in herd health, reducing expenses associated with treatments and culls (23). In particular, the HFSR crossbred has effectively shown a better performance in fertility and longevity than HF in commercial dairy farms (24), and apparently it also achieves a better protective response after BCG vaccination (Table 1).

Recent studies have been showing non-specific effects of BCG in cattle, including the development of a trained immune response in calves subjected to controlled experiments, as well as increased milk production in dairy cows that received the vaccine at 11 months old (11, 12). These recent findings agree with previous reports about several non-specific and beneficial effects of BCG vaccination in people and animals (19, 25).

In this work, our aim was to investigate whether similar outcomes could be observed in productive and sanitary indicators during the initial lactation period of animals that received a single dose of BCG in their early weeks of life, under the conditions of a commercial dairy farm. In this regard, the milk production during first 180 lactation days was compared between groups, although no differences were observed (*p* > 0.05, Supplementary Figure S1), contrasting with the previous Chilean experience with animals vaccinated at 11 month-old (12). This non-specific BCG effect has not been physiologically explained, although it may be related to a metabolic higher efficiency in the glucose utilization, which in humans has been observed as a long-lasting effect (26, 27). Since in this work we did not detect a change in milk yields, we infer that such metabolic influence of BCG lasts lower in the glandular cells of cows or occurs through a different and temporary mechanism.

In terms of the BCG effects in health, our analysis focused on the SCC, clinical mastitis and retained placenta. The SCC is an indicator of mammary gland health status, because represents inflammatory processes and is very well correlated with milk production losses (28). Again, the genetic background of HFRS seems to determine a better or long lasting immunological response after BCG inoculation (Table 2), which also includes the health status of the mammary gland (29). In fact, differences have been identified in the innate immune response to pathogens among cattle breeds (30), which may be linked to polymorphisms in multiple genes, as has been described with cattle resistance to bTB (31).

The CM was another indicator of non-specific response to BCG, which showed a lower incidence in the vaccinated group (Table 3), and specifically in warmer months (*p* = 0.04, OR 0.39). The CM caused by bacterial infections corresponds to a major economic loss in dairy farms, mainly due to diminished milk production, treatment costs and loss of animals (28, 29). Commercial dairy herds from central Chile exhibit a high frequency of *E. coli* and coagulase-negative Staphylococci among CM causing bacteria (32). Nonetheless, various infectious pathogens have been identified, including a wide range of environmental and contagious bacteria that may vary in occurrence throughout the year and in geographic distribution (17, 32). Unfortunately, a relationship between BCG induced immunity and incidental mastitis pathogens cannot be suggested, since etiological diagnosis was not performed to diseased animals in this work, as usually occurs in the dairy farm under study. In addition, it has been reported that the heat stress negatively influences the viability of immune cells in the mammary gland, weakening the immunocompetence of dairy cows (33). Considering that during summer in central Chile the temperatures typically reach 30°C to 35°C around midday, this may also be a seasonal factor modulating the mastitis incidence in animals from this study. And the breed could also be a third variable to analyze in the observed CM frequencies, since the vaccinated HFRS animals were close to significance (*p* = 0.06) when compared with the control group, in accordance with the SCC result, a contrast that was not observed with HF animals (Table 3). Then, more specific analyses should be performed to characterize the differential incidence rates of CM between groups, implying a beneficial non-specific effect of BCG.

The retained placenta was a variable under study because it may be associated to genetic, immunological, metabolic, and infectious disorders causing an incomplete placental maturation (34–36). Despite in this work no significant differences were observed between groups (Table 3), in all analyzed comparisons the RP cases were more frequent in the non-vaccinated group of animals, suggesting the need of more evaluations about the influence of BCG in the development of this pathology. However, when CM and RP were analyzed as a whole, a clear and significant pattern was observed in most scenarios (Figure 1), suggesting that BCG induces a non-specific protective effect in animals, with particular importance during warmer months. A likely mechanism behind this protection may involve the interplay between trained immunity and the gut (37), since the BCG vaccine modifies the intestinal microbiome, altering circulating metabolites and subsequently allowing the development of innate immune memory cells at distal tissues (38, 39).

Non-specific effects of BCG vaccination were not assessed in previous field trials conducted on calves, which involved various breeds and follow-up periods (5, 40–44). The study that observed enhanced milk production as a result of BCG vaccination was conducted on heifers vaccinated at 11 months of age (12), which in the context of this work, it may suggests that this productive effect becomes apparent during around 12 but not 24 months after vaccination. In addition, a post-partum BCG inoculation of cows in an experimental setting failed to identify significant changes in milk production (45), although the age of inoculation, the BCG substrain and its dosage were different. Then, comparisons with previous reports are hindered due to variations in study designs and objectives. Consequently, it is imperative to device experiments that enable the analysis of both the specific and non-specific effects of the BCG vaccine in cattle.

In conclusion, administering the *M. bovis* BCG strain to dairy calves within a commercial dairy herd showed specific and non-specific beneficial effects. It resulted in improved health status of the mammary gland during the initial 180 days of the lactation period, although not modifying milk yields. Both the breed and the season may influence such non-specific response to BCG. The characterization of protection conferred by the BCG against *M. bovis* infection and disease, and its non-specific impacts on immunological or physiological traits under natural breeding conditions of commercial dairy farms, require further studies to adjust and support global initiatives aiming to implement BCG vaccination as a preventive measure against tuberculosis in livestock, especially in low- or middle-income regions where the test and slaughter strategy is not supported by the dairy industry.

## Data availability statement

The original contributions presented in the study are included in the article/Supplementary material, further inquiries can be directed to the corresponding author.

## Ethics statement

The animal studies were approved by Comité Institucional de Cuidado y Uso de Animales (CICUA) (Permit N°22555 VET-UCH), Universidad de Chile. The studies were conducted in accordance with the local legislation and institutional requirements. Written informed consent was obtained from the owners for the participation of their animals in this study.

## Author contributions

CC: Formal analysis, Investigation, Methodology, Writing – original draft. RA-M: Formal analysis, Methodology, Writing – review & editing. MD: Methodology, Writing – review & editing. PÁ: Supervision, Writing – review & editing. RL: Data curation, Supervision, Visualization, Writing – review & editing. PR: Conceptualization, Data curation, Formal analysis, Funding acquisition, Project administration, Writing – review & editing.

## Supplementary material

SUPPLEMENTARY FIGURE S1 Cumulative milk production (L) of BCG vaccinated and control groups during the first 180 lactation days. The records were analyzed according to the calving season [warm (from october to march) and cold (from april to september)], the breed (HF, Holstein Friesian, and HFSR, HF x Swedish Red crossbred F1 generation), and the overall dataset (*p* >0.05).
