# Supplementary figures and images for: Specific and non-specific effects of Mycobacterium bovis BCG vaccination in dairy calves
Source: Front Vet Sci. 2023 Oct 6;10:1278329. doi: 10.3389/fvets.2023.1278329 (PMC10588636; doi:10.3389/fvets.2023.1278329)

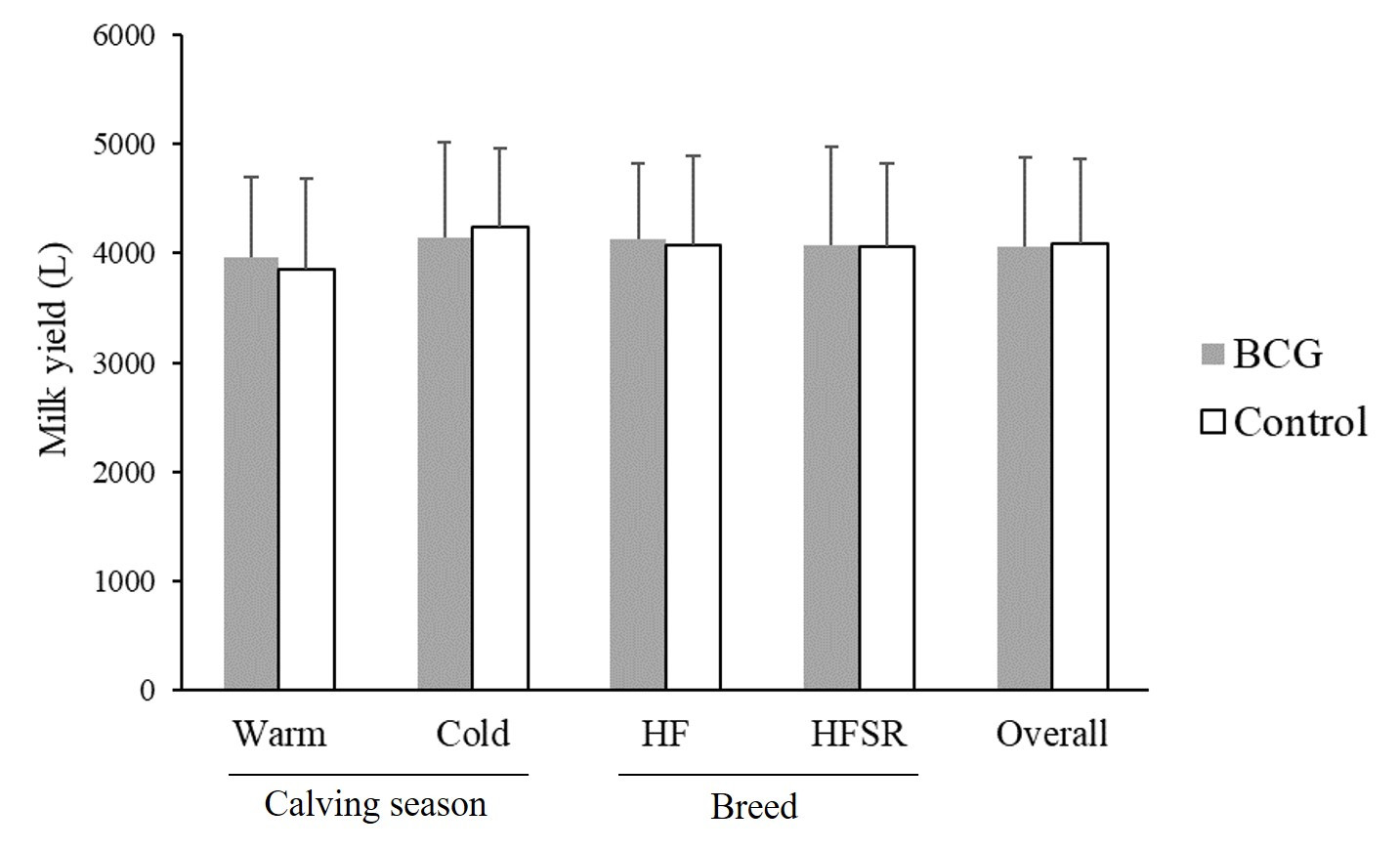

Supplement: SUPPLEMENTARY FIGURE S1 — Cumulative milk production (L) of BCG vaccinated and control groups during the first 180 lactation days. The records were analyzed according to the calving season [warm (from october to march) and cold (from april to september)], the breed (HF, Holstein Friesian, and HFSR, HF x Swedish Red crossbred F1 generation), and the overall dataset (p >0.05). [file Image_1.jpg]
